# Effect of Alloying Elements on the *α*-*γ* Phase Transformation in Iron

**DOI:** 10.3390/ma12081355

**Published:** 2019-04-25

**Authors:** Jerome Meiser, Herbert M. Urbassek

**Affiliations:** Physics Department and Research Center OPTIMAS, University Kaiserslautern, Erwin-Schrödinger-Straße, D-67663 Kaiserslautern, Germany; jmeiser@rhrk.uni-kl.de

**Keywords:** iron, iron alloy, phase transformation, free energy

## Abstract

Small concentrations of alloying elements can modify the α-γ phase transition temperature $T_{c}$ of Fe. We study this effect using an atomistic model based on a set of many-body interaction potentials for iron and several alloying elements. Free-energy calculations based on perturbation theory allow us to determine the change in $T_{c}$ introduced by the alloying element. The resulting changes are in semi-quantitative agreement with experiment. The effect is traced back to the shape of the pair potential describing the interaction between the Fe and the alloying atom.

## 1. Introduction

Solid-solid phase transformations in iron, iron alloys, and steels are relevant in many branches of technology. Modeling of these phase transformations occurs on the level of atomistic (molecular dynamics) simulation [1,2], ab-initio calculations [3], or phase-field simulations [4,5]. For the case of atomistic simulations based on molecular dynamics or Monte Carlo, the interatomic interaction potentials governing the interaction between Fe atoms and between Fe and the alloying atoms is of fundamental importance. Often these potentials are developed with an emphasis on the structural, mechanical and thermophysical properties of the material. However, a fundamental understanding of how an alloying element influences the phase transformation at the level of its interatomic interactions has up until now been lacking.

**Figure 1:**
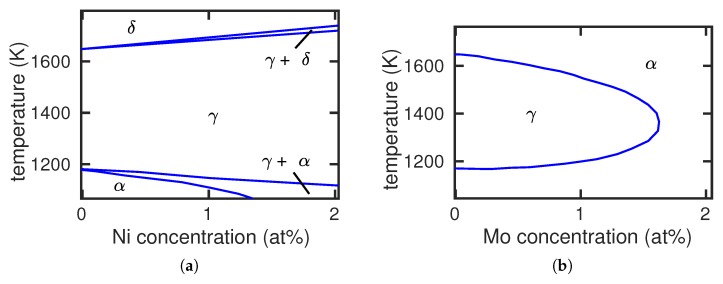
Fe-Ni (**a**) and Fe-Mo (**b**) phase diagrams exemplifying the case of γ and α stabilizing alloying elements, respectively. Experimental data from [6].

Pure Fe at low temperatures has a bcc structure (α phase) but changes to the fcc structure (γ phase) at a temperature of $T_{c}=1183$ K. At an even higher temperature, 1665 K, Fe changes back to the bcc phase, denoted as δ. We shall not be interested in this transformation; rather, we shall focus on the dependence of the α–γ transition temperature on the presence of an alloying element X. If X decreases $T_{c}$, the γ phase grows at the expense of the α phase; such alloying elements are denoted as γ stabilizers (see Figure 1a for the example of Ni). If, on the other hand, $T_{c}$ increases, the γ phase is destabilized and may disappear altogether at higher X concentrations; the corresponding phase diagram is denoted as a γ loop (see Figure 1b for the example of Mo). Such alloying elements are α stabilizers.

Among the many potentials that have been developed for the atomistic modeling of Fe, only few [7,8,9] are able to incorporate the α-γ phase transition as was shown by free-energy studies [10]. Among the alloying elements, C has been modeled with particular care and several potentials have been developed to describe interstitial C in an Fe matrix [11,12,13,14,15,16,17,18,19]; however, these focus on describing the low-temperature bcc phase. Sak-Saracino and Urbassek [20] implemented a pair-potential description of the Fe–C interaction [21,22] and combined it with a phase-transforming Fe potential [7] to study the performance of such potentials to feature the influence of the C concentration on the α-γ phase transition [23].

Ab-initio studies are available to determine the phase diagram of pure Fe [3]. However, the influence of alloy elements on the α-γ phase transition in Fe has only rarely been studied using ab-initio techniques [24].

In this paper, we study the influence of small concentrations of alloying elements on the α-γ phase transition in Fe. To this end, we use a set of potentials that satisfactorily describes the interaction of several elements X with Fe [25]. Using free-energy calculations, we study the influence of the alloying element X on the transition temperature. We find that the results are in good agreement with experimental observations and discuss the pair-potential contribution of the interaction potential in order to identify the cause for the increase or decrease in $T_{c}$ observed.

## 2. Method

### 2.1. Interatomic Interaction Potentials

In order to study the influence of alloying elements on the bcc→fcc phase transformation in Fe, we need interaction potentials of these alloying elements X with Fe. Such a set of potentials has been provided by Zhou et al. [25,26,27] for a set of 15 alloying elements X. In the following, we shall denote this set of potentials as the Johnson potential in view of the long-standing work of Johnson on building potentials [21,28,29].

Unfortunately, the Johnson Fe potential does not feature a bcc→fcc transition for Fe; the bcc phase is stable up to the melting temperature. We therefore modify it to include the bcc→fcc transformation with a transition temperature of 1183 K, as in experiment; the details are reported in Appendix A. We denote this potential as the modified Johnson potential. The results presented in this paper are for this set of potentials.

### 2.2. Free Energies

Let us consider an Fe crystal of *N* atoms with periodic boundary conditions; we denote its free energy as $F^{\mathrm{Fe}}$, and the free energy per atom as $f^{\mathrm{Fe}}=F^{\mathrm{Fe}}/N$. The crystal structure will be indicated by a subscript index. We shall also consider Fe crystals containing (N−1) Fe atoms and 1 alloying atom X in periodic boundary conditions. By varying *N*, this procedure will allow us to study various concentrations of X, c=1/N. We denote the free energy as $F^{\mathrm{Fe}+X}$.

**Figure 2:**
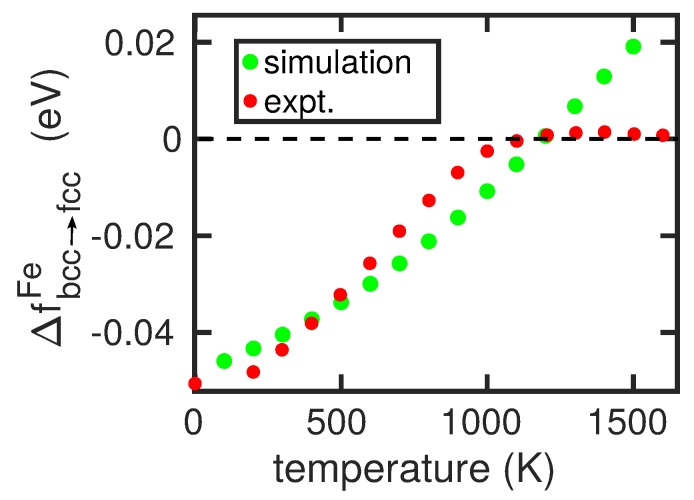
Free-energy difference of the bcc and fcc phase of Fe in the modified Johnson potential presented in Appendix A as a function of temperature. Experimental data from [35].

The bcc→fcc phase transformation in pure Fe is studied with the help of the free-energy difference $\Delta F_{\mathrm{bcc}\to \mathrm{fcc}}^{\mathrm{Fe}}=F_{\mathrm{bcc}}^{\mathrm{Fe}}-F_{\mathrm{fcc}}^{\mathrm{Fe}}$. If it is larger than 0, the fcc phase is stable, otherwise the bcc phase is stable. For an elemental crystal, there exist a number of methods to calculate this quantity, such as the method of metric scaling or thermodynamic integration [10,30,31]. Here, we shall follow the work of Freitas et al. [32] and use the method of λ integration along a Frenkel–Ladd path [33] implemented in LAMMPS [32,34]. Here, we use crystals containing approximately $10^{4}$ atoms and calculate the free energies at integer multiples of 100 K. Figure 2 shows the free-energy difference for the modified Johnson potential. It features a transition temperature of 1183±1 K.

**Figure 3:**
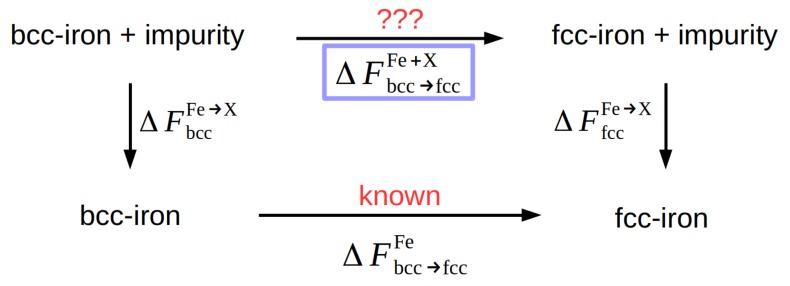
Thermodynamic cycle for the determination of the free-energy difference between the bcc and fcc phase in Fe containing an alloying element X.

It would be disadvantageous to calculate the free energy of an alloyed Fe crystal using the same method since the small changes expected for small concentrations would call for extreme accuracy in the free-energy calculations. Rather, we use the thermodynamic cycle depicted in Figure 3 and calculate the requested quantity $\Delta F_{\mathrm{bcc}\to \mathrm{fcc}}^{\mathrm{Fe}+X}$ from

(1) $$\Delta F_{\mathrm{bcc}\to \mathrm{fcc}}^{\mathrm{Fe}+X}=\Delta F_{\mathrm{bcc}\to \mathrm{fcc}}^{\mathrm{Fe}}+\Delta F_{\mathrm{bcc}}^{\mathrm{Fe}\to X}-\Delta F_{\mathrm{fcc}}^{\mathrm{Fe}\to X}.$$

Here, $\Delta F_{\mathrm{bcc}}^{\mathrm{Fe}\to X}$ denotes the free-energy change when 1 out of *N* Fe atoms is changed to an X atom in a bcc lattice; for an fcc lattice, this quantity is defined analogously. This quantity can be calculated using free-energy perturbation theory [31,36,37]. For a system in which an Fe atom is exchanged to an X atom, Zwanzig’s equation for the free-energy change at temperature *T* gives

(2) $$\Delta F^{\mathrm{Fe}\to X}=-k_{B}T\cdot \ln\langle \exp-\frac{E^{\mathrm{Fe}+X}-E^{\mathrm{Fe}}}{k_{B}T}\rangle ,$$

where $k_{B}$ is Boltzmann’s constant. $E^{\mathrm{Fe}}$ is the potential energy of the pure Fe crystal, $E^{\mathrm{Fe}+X}$ is the potential energy of the alloyed crystal, and 〈⋯〉 indicates the average. This average is determined from 1-ns trajectories in crystals containing N=256 atoms.

We use the perturbation theory only at the transition temperature of pure Fe, 1183 K, since for small concentrations of X, the influence of temperature will be minor.

As an abbreviation, we will use

(3) $$\Delta F_{\mathrm{ex}}=\Delta F_{\mathrm{bcc}}^{\mathrm{Fe}\to X}-\Delta F_{\mathrm{fcc}}^{\mathrm{Fe}\to X}.$$

This quantity gives the difference between the fcc and bcc energies caused by the exchange of an Fe atom by an X atom.

Now, unfortunately, even the Fe atom in the original Johnson parameterization [25] has a non-vanishing $\Delta F_{\mathrm{bcc}\to \mathrm{fcc}}^{\mathrm{Fe}+X}$ in the modified Johnson potential, which we will denote as the offset value $\Delta F_{\mathrm{off}}$. We therefore correct all calculated free-energy differences by this value and finally obtain

(4) $$\Delta F_{\mathrm{bcc}\to \mathrm{fcc}}^{\mathrm{Fe}+X}=\Delta F_{\mathrm{bcc}\to \mathrm{fcc}}^{\mathrm{Fe}}+\Delta F_{\mathrm{ex}}-\Delta F_{\mathrm{off}}.$$

From our simulations, we determined $\Delta F_{\mathrm{off}}=-0.171\pm 0.006$ eV.

### 2.3. Simulation

Simulations are performed with the open-source code LAMMPS [34]. The crystals are set up at 0 K using conjugate gradients for energy minimization. Subsequently, they are relaxed at 5 K for 50 ps before they are heated to the desired temperature with heating rates in the region of 1 K/ps; there they are again relaxed for 50 ps. Temperature control is performed using a Nose-Hoover thermostat [38,39].

The calculations in the pure Fe crystal are performed with N=256 atoms. For the thermodynamic perturbation simulations, we use crystal sizes of N=50–400, corresponding to X concentrations of 0.25–2.0 at%.

The perturbation energy averages in Zwanzig’s equation, Equation (2), are calculated for 10 individual trajectories that differ by using different heating rates and relaxation times to obtain uncorrelated starting configurations.

## 3. Results

The paper by Zhou et al. [25] describes the interaction of 15 elements with Fe. Of these, we could only use seven elements, four of which are known to be γ stabilizers (Pd, Au, Cu, Ni), two of which are α stabilizers (W, Mo), while Co is neutral. For the other elements found in [25], our strategy did not work for the following reasons. (i) Several elements (Pb, Mg, Zr, Ag) are only poorly soluble in Fe, such that strong lattice distortions are generated and the energy of the Fe environment of an alloying atom is considerably changed. (ii) For Ti, Ta, Al, Pt, the interactions between alloying atoms themselves are strong, such that our method—which is based on isolated alloying atoms—cannot determine the interaction at a 1 at% concentration of alloying atoms.

### 3.1. Transition Temperatures

For the remaining elements, in Table 1 we show the free-energy difference $\Delta F_{\mathrm{bcc}\to \mathrm{fcc}}^{\mathrm{Fe}+X}$ at a temperature of 1183 K, i.e., at the phase transition temperature of pure Fe, as determined from Equation (4). We see that the free-energy difference is indeed positive for Pd, Au, Cu, and Ni so that here the γ phase is stabilized, while for W and Mo the α phase is stabilized; these features are in agreement with experiment. Co is a special case; in experiment $T_{c}$ is almost independent of Co concentration up to a Co concentration of 50 at%, while our results predict a weak stabilization of the γ phase. We surmise that this is caused by the fact that the ground-state structure of Co is hcp, in contrast to Pd, Au, Cu, and Ni, which are fcc.

**Figure 4:**
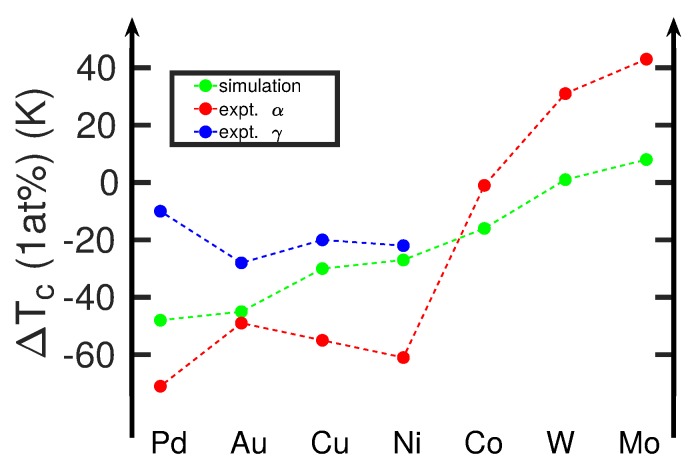
Change in the α-γ transition temperature, $\Delta T_{c}$, induced by various alloying atoms X at 1 at% concentration in Fe. Simulation results are compared to experimental data from [6,40]. Here, α and γ denote the boundaries of the α and the γ field to the coexistence field, α+γ, cf. Figure 1a. If no γ data are shown (Co, W, Mo), the coexistence field is negligible or non-existent.

In order to quantify the changes in the transition temperature, we fit our result for $\Delta F_{\mathrm{bcc}\to \mathrm{fcc}}^{\mathrm{Fe}}$, in Figure 2, to a second order polynomial in *T*:

(5) $$\Delta f_{\mathrm{bcc}\to \mathrm{fcc}}^{\mathrm{Fe}}\left(T\right)=0.0031789\cdot \frac{{\left(T-800\right)}^{2}}{447.21^{2}}+0.020984\cdot \frac{\left(T-800\right)}{447.21}-0.020293,$$

where temperature *T* is in K and the free energy in eV. Using this fit, we can determine the temperatures where the free-energy difference of the alloyed systems, Equation (4), is zero (i.e., the transformation temperatures). Focusing on a concentration of 1 at%, we obtain the results in Figure 4. We can compare them with experimental data of the boundaries of the α and γ fields [6]. Since our free-energy approach only provides a single value for the transition temperature, we compare our data with the boundaries of the α field, as these are available for all alloying elements. The comparison in Figure 4 shows that our calculations are in gross qualitative agreement with experiment in that along the sequence of elements from Pd to Mo, the transition temperature increases. In detail, however, deviations from this trend show up, for example, during the sequence Au-Cu-Ni, where the experimental transition temperature decreases, while the calculated values increase. This deviation points at a failure of the interatomic potentials used [25]. The strong deviation for Ni, as well as the deviation for Co discussed above, may be due to the ferromagnetic interaction of these elements with Fe, which can only incompletely be featured within a classical interatomic potential. This does not, however, explain the (weaker) deviation for Cu.

A systematic disagreement between calculation and experiment is that the simulated changes in transition temperature are smaller than in reality. The origin of this discrepancy can be traced back to the fact that the temperature-dependence of our free-energy curve for pure Fe, shown in Figure 2, is too steep in comparison to reality [35]. This is caused by the fact that in reality, the free energy must must have a maximum and then decrease to zero again at 1665 K in order to incorporate the transition to the fcc δ phase, see Figure 2. Hence, our simulated changes in transition temperature are smaller than in reality.

**Figure 5:**
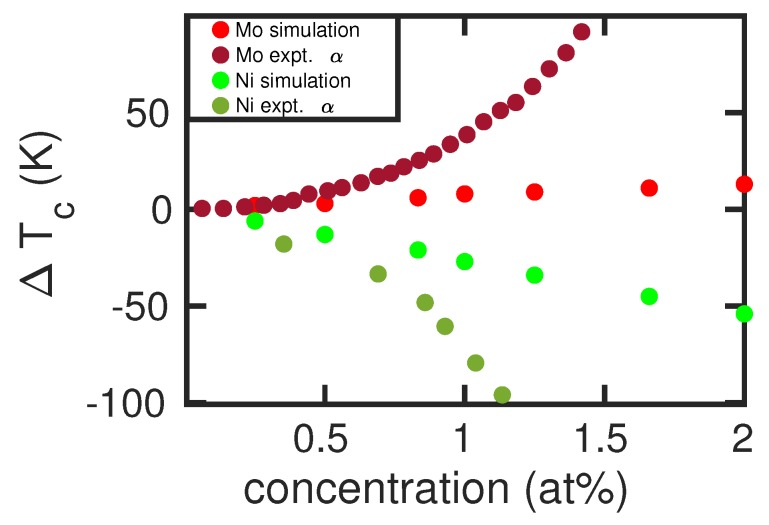
Change in the α-γ transition temperature, $\Delta T_{c}$, induced by Mo and Ni atoms in Fe as a function of concentration of the alloying elements. Simulation results are compared to experimental data [6] of the boundary of the α field.

By changing the size of the simulation crystallite that hosts the alloying atom, the effect of alloying-atom concentration can be studied up to a value of 2 at%; at larger concentrations, X–X interactions become important. Figure 5 shows our results for two cases, an α stabilizer (Mo) and a γ stabilizer (Ni). We see that our results predict an almost linear dependence of the transition temperature on the concentration of the alloying elements, while the experimental data show a larger curvature. The stronger increase of $T_{c}$ with the concentration of the alloying elements in experiment is again caused by the too steep $\Delta F_{\mathrm{bcc}\to \mathrm{fcc}}^{\mathrm{Fe}}\left(T\right)$ curve discussed above, see Figure 2. Thus, even large changes in free energy (induced by the alloying atoms) translate to only small changes in $T_{c}$.

### 3.2. Connection to Potentials

In order to understand which features of the potential contribute to the changes in the free energy—and thus the transition temperature—caused by the alloying elements, we focus on the pair-potential contribution $\psi_{\mathrm{FeX}}\left(r\right)$ (see Appendix A). In embedded-atom-model potentials, besides the pair-potential contribution, the embedding energy in the electron density donated by the surrounding atoms also contributes to the potential energy; however, this energy changes less around an alloying atom than the pair-potential contribution since the embedding energy of Fe has a minimum at the equilibrium electron density. The total Fe–X pair contribution originates from a summation over all Fe neighbors of the alloying atom X,

(6) $$\Delta E_{\mathrm{pair}}=\sum_{i}N_{i}^{\mathrm{bcc}}\cdot \psi_{\mathrm{FeX}}\left(r_{i}^{\mathrm{bcc}}\right)-\sum_{j}N_{j}^{\mathrm{fcc}}\cdot \psi_{\mathrm{FeX}}\left(r_{j}^{\mathrm{fcc}}\right),$$

where $r_{i}^{\mathrm{bcc}}$ and $r_{i}^{\mathrm{fcc}}$ are the distances, and $N_{i}^{\mathrm{bcc}}$ and $N_{i}^{\mathrm{fcc}}$ are the number of nearest neighbors in the *i*th nearest-neighbor shell of the bcc and fcc phases of Fe, respectively, at room temperature. $\Delta E_{\mathrm{pair}}$ compares the pair-potential part of the Fe–X interaction in the bcc and fcc phases. $\psi_{\mathrm{FeX}}\left(r\right)$ is calculated according to Equation (A3).

**Figure 6:**
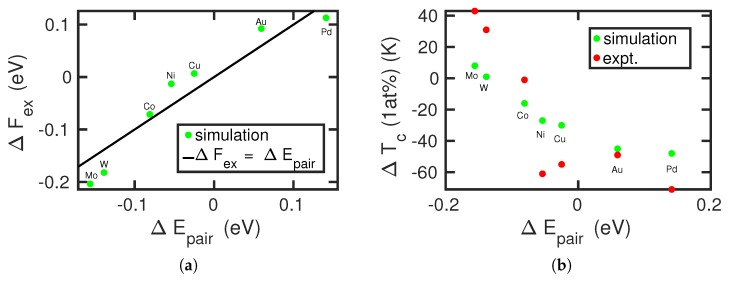
Correlation of (**a**) the energy difference between the fcc and bcc structures caused by the exchange of an Fe atom by an alloying atom, $\Delta F_{\mathrm{ex}}$, Equation (3), and (**b**) the change in α–γ transition temperature, $\Delta T_{c}$, with the pair energy difference $\Delta E_{\mathrm{pair}}$, Equation (6). Experimental data in (**b**) are from [6,40]. The line in (**a**) shows the equality $\Delta F_{\mathrm{ex}}=\Delta E_{\mathrm{pair}}$.

In Figure 6a, we compare the free-energy difference $\Delta F_{\mathrm{bcc}\to \mathrm{fcc}}^{\mathrm{Fe}+X}$ (at 1183 K) with the pair energy difference $\Delta E_{\mathrm{pair}}$. A good correlation is seen, which emphasizes that the pair-potential contribution is responsible for the observed trends in the free energy. The correlation of the transition temperatures with experimental data is shown in Figure 6b. As in Figure 4, the overall trend of the calculated data reproduces that of the experimental data. Marked deviations show up in particular for Ni and Cu, where the pair-potential-energy difference $\Delta E_{\mathrm{pair}}$ is around zero, while experiment shows a strong decrease in the transition temperature as compared to pure Fe. As discussed above, the origin of this discrepancy must be assumed to lie in details of the interatomic interaction potentials.

**Figure 7:**
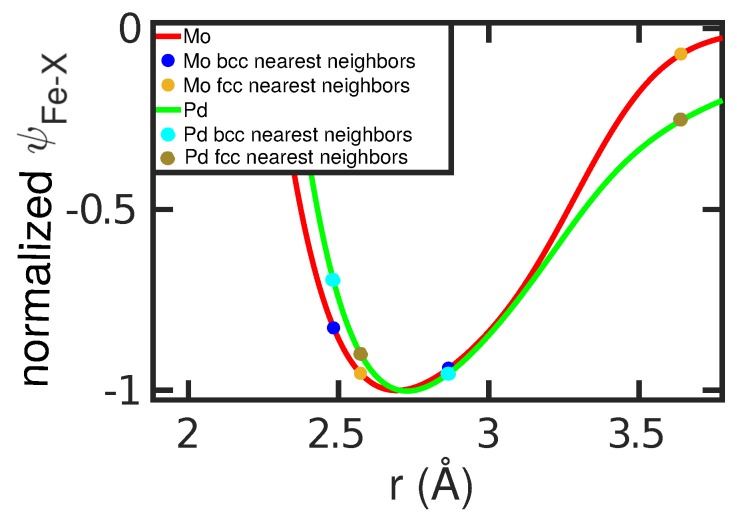
Pair potential of the Fe–X interaction, $\psi_{\mathrm{FeX}}$, for Mo and Pd atoms, normalized to its value at the minimum, $r_{\min}$. The first and second nearest-neighbor distances in the Fe bcc and fcc lattices are marked.

We discuss this good correlation by analyzing the spatial dependence of $\psi_{\mathrm{FeX}}\left(r\right)$ for Mo (α stabilizing) and Pd (γ stabilizing)seen in Figure 7. For these two elements, the two nearest-neighbor distances in the fcc and bcc structures are marked. For Mo, the first nearest neighbors in the bcc structure have considerably lower energy than in Pd; this stabilizes the bcc structure for Mo. For Pd, in addition the second nearest neighbors in the fcc structure have lower energy than in Mo; this helps stabilize the fcc structure in Pd. Since the interspecies potential $\psi_{\mathrm{FeX}}\left(r\right)$ enters the free-energy calculations, this discussion helps to understand how free energies, and hence transition temperatures, are influenced by the interspecies potential.

## 4. Conclusions

Using the potential set of Zhou et al. [25], we investigated the influence of various alloying elements on the α-γ phase transformation in iron. As a first step, the Fe–Fe interaction had to be modified to include the α-γ phase transformation since the original potential does not feature it.

This set of potentials allows us to investigate the admixture of seven different metal species into Fe. Using free-energy calculations based on perturbation theory allowed us to determine the change in $T_{c}$ introduced by the alloying element. The trends of raising or lowering the transformation temperature obtained are in qualitative agreement with experiment.

Our results could be rationalized by studying the pair contribution of the interspecies potential; in this approach [25], it is obtained as the arithmetic mean of the Fe–Fe and the X–X interactions. X–X potentials that stabilize the bcc structure—by providing low-energy nearest-neighbor bcc sites—increase the transformation temperature, while potentials that stabilize the fcc structure—by low energies for second nearest-neighbor fcc sites—raise $T_{c}$.

Our results may prove useful in designing potentials for iron alloys that feature the α-γ phase transformation. The potentials presented here are not yet useful for realistic studies—in particular because their elastic moduli are too large—and have been introduced here for demonstration purposes only. The development of potentials for future studies of the phase transformation behavior of dilute iron alloys would be welcome.

## Figures and Tables

**Table 1:** Difference between the fcc and bcc energies caused by the exchange of an Fe atom by an alloying atom, $\Delta F_{\mathrm{ex}}$, Equation (3), and free-energy difference between the bcc and fcc structures in the alloy, $\Delta F_{\mathrm{bcc}\to \mathrm{fcc}}^{\mathrm{Fe}+X}$, Equation (4), at a temperature of 1183 K and a concentration of 0.39 at%.

	$\Delta F_{\mathrm{ex}}$ (eV)	Error (eV)	$\Delta F_{\mathrm{bcc}\to \mathrm{fcc}}^{\mathrm{Fe}+X}$ (eV)
Pd	0.113	0.006	0.287
Au	0.092	0.007	0.267
Cu	0.007	0.008	0.181
Ni	−0.019	0.006	0.155
Co	−0.071	0.005	0.103
Fe	−0.171	0.006	0.000
W	−0.182	0.006	−0.008
Mo	−0.208	0.007	−0.034

**Figure A1:**
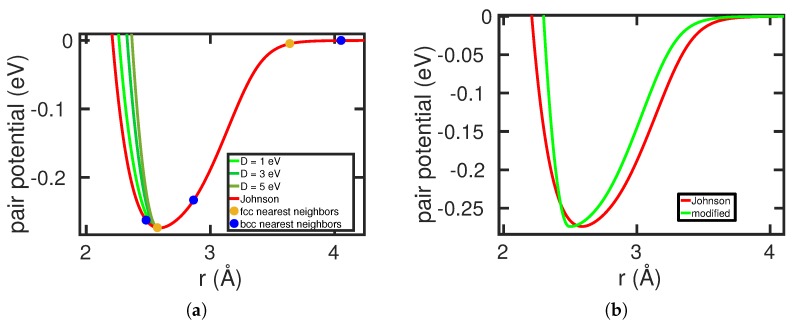
Modification of the Johnson potential [25] for the Fe–Fe interaction, Equation (A5). (**a**) Effect of the modification parameter *D*. The nearest-neighbor positions in bcc and fcc Fe are marked. (**b**) Comparison of the original with the modified Johnson potential.

## Appendix A. The Modified Johnson Potential

The Johnson potential [25] is of the embedded-atom-model form. The pair-potential part of the interaction between two atoms of the same species is given by

(A1) $$\psi \left(r\right)=A\cdot \frac{e^{-\alpha (r/r_{e}-1)}}{1+{\left(r/r_{e}-\kappa \right)}^{20}}-B\cdot \frac{e^{-\beta (r/r_{e}-1)}}{1+{\left(r/r_{e}-\lambda \right)}^{20}}.$$

The electron density is given by

(A2) $$f\left(r\right)=f_{e}\cdot \frac{e^{-\beta (r/r_{e}-1)}}{1+{\left(r/r_{e}-\lambda \right)}^{20}}.$$

The pair potential between different species N and M is constructed as

(A3) $$\psi_{\mathrm{NM}}\left(r\right)=\frac{1}{2}\cdot \left(\frac{f_{M}\left(r\right)}{f_{N}\left(r\right)}\cdot \psi_{\mathrm{NN}}\left(r\right)+\frac{f_{N}\left(r\right)}{f_{M}\left(r\right)}\cdot \psi_{\mathrm{MM}}\left(r\right)\right).$$

The embedding energy functions are given in three different electron density ranges by

(A4) $$F\left(\rho \right)=\left\{\begin{array}{cc} \sum_{i=0}^{3}F_{ni}{\left(\frac{\rho }{0.85\cdot \rho_{e}}-1\right)}^{i}, & \rho <0.85\cdot \rho_{e},\\ \sum_{i=0}^{3}F_{i}{\left(\frac{\rho }{0.85\cdot \rho_{e}}-1\right)}^{i}, & 0.85\cdot \rho_{e}<\rho <1.15\cdot \rho_{e},\\ F_{e}\cdot \left[1-\ln{\left(\frac{\rho }{\rho_{s}}\right)}^{\eta }\right]\cdot {\left(\frac{\rho }{\rho_{s}}\right)}^{\eta }, & \rho >1.15\cdot \rho_{e}, \end{array}\right.$$

where the parameter $\rho_{e}$ describes the equilibrium electron density.

The potential parameters *A*, *B*, α, β, κ, λ, $r_{e}$, $f_{e}$, $F_{ni}$, $F_{i}$, $F_{e}$, $\rho_{e}$, $\rho_{s}$, and η for all 16 materials are tabulated in [25].

Unfortunately, the potential for Fe stabilizes the bcc phase for all temperatures up to the melting point. We therefore modify it so as to feature a phase transition to the fcc phase at a temperature of 1183 K, in agreement with experiment. We do this by only modifying the pair interaction term for the Fe–Fe interaction as

(A5) $$\psi_{\mathrm{mod}}\left(r\right)=\psi \left(r\right)+D\cdot \frac{{\left[r-\left(r_{e}+\Delta r\right)\right]}^{2}}{1+{\left[r/\left(r_{e}+\Delta r\right)\right]}^{1000}}.$$

Here $r_{e}$ is the equilibrium distance between nearest neighbors in the unmodified potential, Equation (A1), and Δr=0.0918 Å is the difference between the nearest-neighbor distances of Fe atoms of the bcc and the fcc phase. The denominator in Equation (A5) has been introduced to cut off the modification at $r_{e}+\Delta r$. The idea behind this modification is to increase the potential at the nearest-neighbor site of the bcc phase in order to destabilize this phase so that the free energy of the bcc phase is no longer lower than the γ phase at all temperatures (see Figure A1a).

We find that D=6.05 eV and $r_{e}=2.3990$ Å reproduce the experimental values of $T_{c}$ and the lattice constants of 2.8656 (3.6041) Å in the bcc (fcc) phase at 300 K [41,42]. Figure A1b compares the original and the modified Johnson potential for the Fe–Fe interaction. We note that while this potential has its merits as a model potential to study the phase transformation in dilute Fe alloys, it has the drawback that the increased curvature at the potential minimum also increases the elastic moduli. The bulk modulus rises from 166 GPa for the Johnson potential to 577 GPa, as compared to the experimental value of 178 GPa.
